# Identification and molecular characterization of the second *Chlamydomonas gun4* mutant, *gun4-II*

**DOI:** 10.12688/f1000research.2-142.v2

**Published:** 2013-07-29

**Authors:** Phillip B Grovenstein, Darryel A Wilson, Kathryn D Lankford, Kelsey A Gaston, Surangi Perera, Mautusi Mitra

**Affiliations:** 1 Department of Biology, University of West Georgia, Carrollton GA, 30118, USA; 2 Current address: Pediatric Infectious Diseases, Emory-Children's Center, Atlanta GA, 30322, USA; 3 Current address: Joseph J. Zilber School of Public Health, University of Wisconsin-Milwaukee, Milwaukee WI, 53204, USA

## Abstract

The green micro-alga *Chlamydomonas* *reinhardtii* is an elegant model organism to study oxygenic photosynthesis. Chlorophyll (Chl) and heme are major tetrapyrroles that play an essential role in photosynthesis and respiration. These tetrapyrroles are synthesized via a common branched pathway that involves mainly enzymes, encoded by nuclear genes. One of the enzymes in the pathway is Mg chelatase (MgChel). MgChel catalyzes insertion of Mg ^2+^ into protoporphyrin IX (PPIX, proto) to form Magnesium-protoporphyrin IX (MgPPIX, Mgproto), the first biosynthetic intermediate in the Chl branch. The GUN4 (genomes uncoupled 4) protein is not essential for the MgChel activity but has been shown to significantly stimulate its activity. We have isolated a light sensitive mutant, *6F14*, **by random DNA insertional mutagenesis. *6F14* cannot tolerate light intensities higher than 90-100 μmol photons m ^-2 ^s ^-1^. It shows a light intensity dependent progressive photo-bleaching. *6F14* is incapable of photo-autotrophic growth under light intensity higher than 100 μmol photons m ^-2 ^s ^-1^. PCR based analyses show that in *6F14* the insertion of the plasmid outside the *GUN4* locus has resulted in a genetic rearrangement of the *GUN4* gene and possible deletions in the genomic region flanking the *GUN4* gene. Our *gun4* mutant has a Chl content very similar to that in the wild type in the dark and is very sensitive to fluctuations in the light intensity in the environment unlike the earlier identified *Chlamydomonas gun4 *mutant. Complementation with a functional copy of the *GUN4 *gene restored light tolerance, Chl biosynthesis and photo-autotrophic growth under high light intensities in *6F14*. *6F14* is the second *gun4* mutant to be identified in *C. reinhardtii*. Additionally, we show that our two *gun4* complements over-express the GUN4 protein and show a higher Chl content per cell compared to that in the wild type strain.

## Introduction

*Chlamydomonas reinhardtii* is a green micro-alga that can grow either heterotrophically using exogenous acetate as a carbon source or photo-autotrophically, using atmospheric CO _2_. It possesses a photosynthetic apparatus very similar to higher plants, has a short and simple haplontic life cycle, can synthesize Chl both light dependently and light independently (unlike most angiosperms) and its genome has been sequenced ^1^. In addition, well developed molecular tools exist for genetic manipulations of its genome. All these traits make this alga an elegant model system for dissecting oxygenic photosynthesis ^2, 3^.

**Figure 1.**
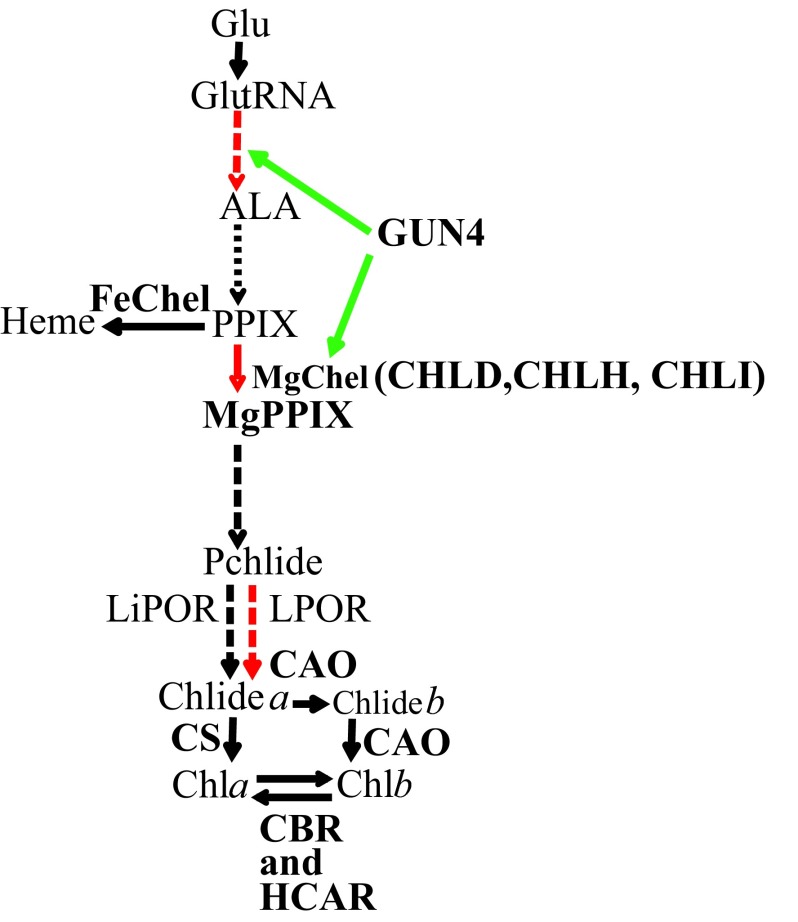
A simplified tetrapyrrole biosynthetic pathway. Light regulated steps are in red. Dashed arrows denote multiple enzymatic steps and green arrows point to steps that are positively regulated by the GUN4 protein, respectively. Tetrapyrrole intermediates and enzymes are shown in black and bold black type, respectively. Readers are advised to look in the text for full names of tetrapyrrole intermediates and enzymes, which are abbreviated in this figure.

Chl, heme, siroheme, cobalamin, heme *d1* and factor F430 are major tetrapyrroles that are involved in wide variety of essential life processes in all living organisms. Chl and heme are synthesized via a common branched pathway ^4, 5^ (outlined in Figure 1). Photosynthetic eukaryotes synthesize 5-aminolevulinic acid (ALA) from glutamine (Glu) bound to tRNA ^Glu^ through the C5 pathway consisting of two steps catalyzed by glutamyl-tRNA reductase and glutamate-1-semialdehyde aminotransferase ^4, 5^. ALA is subsequently converted in six steps to PPIX, the last common precursor for both Chl and heme biosynthesis ^4, 5^. Insertion of Fe ^2+^ into PPIX by ferrochelatase (FeChel) leads to heme. Insertion of Mg ^2+^ in PPIX by the heterotrimeric MgChel (comprised of three subunits: CHLD, CHLH and CHLI ^6^) leads to MgPPIX, the first biosynthetic intermediate in the Chl branch ^6^. MgPPIX is converted to Pchlide via three enzymatic steps. The reduction of Pchlide to form chlorophyllide (Chlide) can occur by two different mechanisms. One mechanism is catalyzed by the strictly light dependent enzyme NADPH:Pchlide oxidoreductase (LPOR) and occurs in all photosynthetic organisms; it is the only mechanism of Chl formation in angiosperms ^7– 10^. The second mechanism is catalyzed by the light independent NADPH:Pchlide oxidoreductase (LiPOR) and is present in anoxygenic bacteria, alga, ferns and gymnosperms ^11– 20^. The Chlide *a* undergoes a phytylation reaction, catalyzed by Chl synthase (CS), resulting in the formation of Chl *a*. In vascular plants and green algae a portion of the Chlide *a* is converted to Chlide *b* by Chlide *a* oxygenase (CAO) prior to phytylation ^21– 24^. Chl *a* is converted to Chl *b* by CAO via formation of 7-hydroxymethyl chlorophyll *a* (HCA) and Chl *b* can be converted back to Chl *a* via HCA by chlorophyll *b* reductase (CBR) and 7-hydroxymethyl chlorophyll *a* reductase (HCAR) ^25^. This inter-conversion of Chl *a* and Chl *b*, referred to as the “chlorophyll cycle”, plays an important role in greening, acclimation to light and senescence ^25^.

Stringent control of tetrapyrrole biosynthesis is especially essential for oxygenic photosynthetic organisms that are often prone to oxidative stress. Free Chl, heme and their immediate precursors are highly photo-toxic molecules and generate reactive oxygen species (ROS) under aerobic conditions ^26^. Hence most of the cellular Chls are usually bound to the light harvesting complex (LHC) and other photosystem (PS) proteins. Chl is made in the plastid. Most of these Chl binding proteins and enzymes of the tetrapyrrole biosynthetic pathways are encoded by the nuclear genes ^5^. Hence a tight coordination of biosynthesis of Chl with its apoprotein is necessary ^27^. Chl and heme biosynthesis in plants is under transcriptional, translational and post-translational control at multi level and is accomplished by a complex regulatory network among the chloroplasts, mitochondria and nucleus, that is not well understood ^28– 30^.

One of the major research interests of our laboratory is to identify components that play a role in the regulation of Chl biosynthesis under different irradiance conditions. We have generated a random DNA insertional *Chlamydomonas* mutant library and have screened it to isolate twenty one mutants that are either defective in Chl biosynthesis and/or are incapable of photo-autotrophic growth under different irradiance conditions. One of the isolated mutants ( *6F14*) is a light sensitive mutant which shows a light intensity dependent progressive photo-bleaching and is incapable of photosynthesis under low light intensities (90–100 µmol m ^-2^ s ^-1^). Molecular analyses revealed that *6F14* is defective in the *GUN4* (genome uncoupled 4) gene which codes for a protein that stimulates MgChel activity. *6F14* is the second *gun4* mutant ( *gun4-II*) to be identified in *Chlamydomonas* ^31^. Transformation of *6F14* with a functional copy of the *GUN4* gene restored the wild type phenotype. Western analyses show that the two isolated *gun4-II* complements are over-expressing the GUN4 protein. Chl analyses show that these *gun4-II* complements have 50–60% more Chl than that of the wild type strain. In this study, we present our molecular data on the identification of the mutation locus in *6F14* and its complementation.

## Materials and methods

### Algal media and cultures

*Chlamydomonas* strains 4A+ (a gift from Dr. Krishna Niyogi (UC, Berkeley), *gun4-II* and *gun4-II* complements (both generated by our laboratory) were grown either in Tris-Acetate Phosphate (TAP) heterotrophic media or in Sueoka’s High Salt (HS) photo-autotrophic media. TAP and HS liquid media and agar plates were prepared in the lab using reagents from Fisher Scientific (Pittsburgh, PA) according to the protocol given in Gorman and Levine (1965) ^32^ and Sueoka (1960) ^33^, respectively. The 4A+ strain and *gun4-II* complements were maintained on TAP agar plates and TAP + zeocin (Sigma, St. Louis, MO) plates, respectively under dim light intensities (10–15 µmol photons m ^-2^ s ^-1^) at 25°C. The final zeocin concentration was 15 µg/ml. The *gun4-II* mutant ( *6F14*) was maintained in the dim light or in the dark on TAP 1.5% agar plates containing 10 µg/ml of paromomycin (Sigma, St. Louis, MO). Liquid algal cultures used for RNA and genomic DNA extractions and protein analyses were grown in 100 ml flasks on the New Brunswick Scientific Excella E5 platform shaker (Enfield, CT) in TAP media at 150 rpm in the dim light.

### Generation of the *6F14* mutant

**Figure 2.**
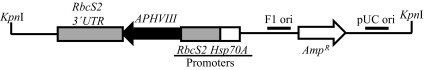
Linearized pBC1 plasmid used for random insertional mutagenesis. The cleavage site of *Kpn*1 restriction enzyme, used for linearization of the vector is shown. *APHVIII* is under the control of combo promoters which consist of the promoter of the gene encoding the small subunit of Rubisco (RbcS2) and the gene encoding the heat shock protein 70A (Hsp70A). pBC1 is a phagemid and its F1 origin (F1 ori) and pUC origin (pUC ori) are shown. The size of the plasmid is 4763 bp.

The purified pBC1plasmid from the DH5α *Escherichia coli-pBC1* clone (obtained from Dr. Krishna Niyogi’s laboratory at UC, Berkeley) was used for random DNA insertional mutagenesis. This plasmid contains two antibiotic resistance genes: *APHVIII* and *Amp ^R^* ( Figure 2). *APHVIII* confers resistance against the antibiotic paromomycin and was used as a selection marker for screening of *Chlamydomonas* transformants. *Amp ^R^* was used as a selection marker for screening of *E. coli* clones harboring the pBC1 plasmid. *E. coli* was grown in 1 l of Luria Bertani (LB) broth containing 1% tryptone, 0.5% of yeast extract, 1% NaCl and ampicillin (final concentration of ampicillin:100 µg/ml). LB media was prepared in the laboratory using reagents purchased from Fisher (Pittsburgh, PA). Ampicillin was purchased from Fisher (Pittsburgh, PA). The culture was incubated at 37°C overnight. Plasmid purification from *E. coli* cells was facilitated by a Qiagen plasmid mega kit according to the protocol given in the technical manual (Qiagen, Valencia, CA). Once purified from *E. coli*, the circular pBC1 vector was linearized with the restriction enzyme *Kpn*I (NEB, Beverly, MA) according to the protocol given in the technical manual. The linearized DNA was purified using a QIAEX II gel extraction kit (Qiagen, Valencia, CA) according to the protocol given in the technical manual. All agarose DNA gel electrophoresis was visualized by BioRad Molecular Imager Gel Doc XR+ (BioRad, Hercules, CA). Transformation of parental strain 4A+ by the linearized pBC1 vector was performed utilizing the glass bead transformation technique described by Kindle *et al.* (1989) ^34^ and Dent *et al.* (2005) ^2^. Transformants were plated onto fresh TAP agar plates containing 10 µg/ml paromomycin (TAP+P) in the dark. Single colonies of mutants were picked and transferred onto fresh TAP+P plates using a numbered grid layout. Screening of photosynthetic and pigment deficient mutants was done by visual inspection and monitoring of growth under different light intensities in heterotrophic, mixotrophic and photo-autotrophic conditions ^2^.

### Genomic DNA and RNA extraction

4A+, *gun4-II* complements and *gun4-II* were grown in TAP liquid media in the dim light to a cell density of about 5 × 10 ^6^ cells/ml of the culture. Genomic DNA was purified using a phenol-chloroform extraction method ^35^. RNA extraction was facilitated by TRIzol reagent from Invitrogen (Carlsbad, CA) following the protocol in the technical manual. DNA and RNA concentrations were measured using a Nanodrop 1000 spectrophotometer from Thermo Fisher Scientific (Wilmington, DE). DNase treatment was performed using Ambion’s TURBO DNA-free kit from Invitrogen (Carlsbad, CA) following the protocol in the technical manual to remove genomic DNA from the RNA preparation. Generation of cDNA was performed using Life Technologies Superscript III First-Strand Synthesis System from Invitrogen (Carlsbad, CA) following the protocol in the technical manual.

### Thermal Asymmetric InterLaced PCR

TAIL (Thermal Asymmetric InterLaced) PCR was implemented, following the protocol of Dent *et al.* (2005) ^2^. HotStar Taq Plus DNA polymerase kit reagents (Qiagen, Valencia, CA) were used for PCR. The PCR reaction mixture consisted of 1 × PCR buffer, 200 µM of each dNTP, 1 × Q-solution, 2.5 units of HotStar Taq Plus DNA polymerase, 60 pmoles of the random degenerate primer RD1 and 5 pmol of the *APHVIII* specific primer. Primers were ordered from IDT (Skokie, IL; Table 1). Degenerate primer RD1 has an average *T*_m_ of 51°C while the three *APHVIII* specific primers used had *T*_m_ ranging from 58°C to 64°C. PCR cycling programs were created using the program given in Dent *et al.* (2005) ^2^. TAIL1 PCR product was diluted 10-fold and 2 µl of the diluted TAIL1 PCR product was used for TAIL2 PCR reactions. The TAIL2 PCR product was gel purified using a QIAEX II gel extraction kit (Qiagen, Valencia, CA) according to the protocol given in the technical manual. Purified TAIL2 PCR product was sequenced at the UC, Berkeley DNA Sequencing Facility (Berkeley, CA). All primer sequences are shown in Table 1.

**Table 1.** List of primers used for TAIL (Thermal Asymmetric InterLaced) PCR, verification of TAIL PCR product and DNA sequencing. These primers were used to generate the data in Figure S2 and Figure S3.

Primer name	Sequence of primer	Location
RD1	5´-WNG GGS CNG CWT TT-3´	Degenerate primer
7F	5´-ACG GAG GAT CGT TAC AAC CAA CAA-3´	*APHVIII* 3´ UTR
2R	5´-CTC AAG TGC TGA AGC GGT AGC TTA-3´	*APHVIII* 3´ UTR
3R	5´-TCT TCT GAG GGA CCT GAT GGT GTT-3´	*APHVIII* 3´ UTR
4R	5´-GGG CGG TAT CGG AGG AAA AGC TG-3´	*APHVIII* 3´ UTR

### Genomic and reverse transcription PCR

Primers were designed based on genomic DNA sequences available in the *Chlamydomonas* genome database in Phytozome. Amplifications of genomic DNA and cDNA were executed using the MJ Research PTC-200 Peltier Thermal Cycler (Watertown, MA). HotStar Taq Plus DNA polymerase kit (Qiagen, Valencia, CA) was used for PCR following the cycling conditions given in the Qiagen protocol booklet. Annealing temperature was between 55 and 60°C depending on the *T*_m_ of the primers. Extension time was varied according to the size of the PCR product amplified. Final extension was set at 72°C for ten minutes. All genomic and reverse transcription PCR products were amplified for a total of thirty-five cycles. A 50–150 ng sample of genomic DNA or cDNA were used for PCR reactions. For semi-quantitative RT-PCR reactions, 3 µg of total RNA was converted into cDNA and then 150 ng of cDNA templates were used for RT-PCR. Sequences of primers used for genomic and RT-PCR are shown in Table 2– Table 4.

**Table 2.** List of *GUN4* specific primers. These primers were used for *GUN4* (Cre05.g246800) genomic DNA PCR on *6F14* and 4A+ and also for DNA sequencing to generate the data in Figure S3 and Figure S4.

Primer name	Sequence of primer	Location
2R	5´-AGTGTGTGTTTGGGCCAGCATTT-3´	Exon1
3F	5´-TGTGGAGAAGAAGAAGTCCGGCAA-3´	Exon1
3R	5´-TTGCCGGACTTCTTCTTCTCCACA-3´	Exon1
14F	5´-GATCCGCAGCCTCACGAG-3´	Exon1
14R	5´-CCTCGTGAGGCTGCGGATC-3´	Exon1
7F	5´-ACAACCCTTGACTTGCGACTCTGT-3´	Exon2
7R	5´-ACAGAGTCGCAAGTCAAGGGTTGT-3´	Exon2
8F	5´-ACCGCATCTTGCAAAGATTGCACC-3´	Exon2
8R	5´-GGTGCAATCTTTGCAAGATGCGGT-3´	Exon2
10R	5´-AGTCTTACACAGGCATACTGCAGCG-3´	Exon2
11R	5´-CTCTTTCAGTCTTACACAGGCATACTGC-3´	Exon2
12F	5´-AGCCGGACTGTTGCGTAATGTGAT-3´	Exon2
12R	5´-ATCACATTACGCAACAGTCCGGCT-3´	Exon2

**Table 3.** List of primers used for checking the genomic region upstream of *GUN4* (Cre05.g246800) and *HYP2* [g5195] gene. These primers were used to generate the data in Figure S5 and Figure 6.

Primer name	Sequence of primer	Location
ACF6	5´-ACATAGCAGCGAGACACACCACAT-3´	Upstream of *GUN4* region
ACF7	5´-AACAAATCCGCGAACGCCACTATG-3´	Upstream of *GUN4* region
ACR7	5´-CATAGTGGCGTTCGCGGATTTGTT-3´	Upstream of *GUN4* region
ACF11	5´-GCAACCGGTGTTTGGGCGTATTAT-3´	Upstream of *GUN4* region
ACR11	5´-ATAATACGCCCAAACACCGGTTGC-3´	Upstream of *GUN4* region
H3F	5´-TCCCATGGTATCCCGAGCTTGAAA-3´	3´ end of *HYP2*
H4F	5´-TGAGGAAACTGGACTTGGCTGAGT-3´	3´ end of *HYP2*
H5F	5´-TACCAGCAGCATCTAAGCACCACA-3´	3´ end of *HYP2*
H6R	5´-TATTCTAATGCAGCACGGCAAGGC-3´	3´ end of *HYP2*

**Table 4.** List of primers used for transcript analysis of *GUN4* and *GUN4* neighboring genes in *6F14*. These primers were used to generate the data in Figure 7. The gene loci numbers in Phytozome for the three neighboring genes of *GUN4* on chromosome 5 and the control actin gene on chromosome 13 are: *HYP1* [Cre05.g246750], *HYP2* [g5195] and *SOXE* [Cre05.g246900] and *Actin* (Cre13.g603700), respectively.

Primer name	Sequence of primer	Purpose
F2	5´-ACGACACCACCTTCAACTCCATCA-3´	Actin
R2	5´-TTAGAAGCACTTCCGGTGCACGAT-3´	Actin
NupF4	5´-TGTATGAACTCTGAGCAGGCGACA-3´	*HYP1*
Nup98R2	5´-CCTGCCGTATGTCGTGCACAAAC-3´	*HYP1*
3F	5´-TGTGGAGAAGAAGAAGTCCGGCAA-3´	*GUN4*
8R	5´-GGTGCAATCTTTGCAAGATGCGGT-3´	*GUN4*
HypF2	5´-TTCCTGGCTACTGCCGTATTCGCA-3´	*HYP2*
H6F	5´-GCCTTGCCGTGCTGCATTAGAATA-3´	*HYP2*
PB120	5´-GCACGGATGGCAAGTACATG-3´	*SOXE*
PB121	5´-CTACTTCACTGCCCTGGAGTTT-3´	*SOXE*

### Cloning of the *GUN4* gene in the pDBle vector

The pDBle vector (obtained from Dr. Saul Purton, University College London, UK) was double-digested with restriction enzymes *Eco*RI and *Nde*I (NEB, Beverly, MA) according to the protocol given in the technical manual. The *GUN4* gene was amplified using primers given in Table 5. Ligation of the double digested ( *Nde*I and *Eco*RI digested) *GUN4* gene and the *Nde*I/ *Eco*RI double-digested pDBle vector was done using the T4 ligase and 1 mM ATP (NEB, Beverly, MA). Chemically competent (CaCl _2_ treated) *E. coli* cells were used for transformation. After transformation, *E. coli* cells were plated on LB+ampicillin (final concentration of ampicillin:100 µg/ml) plates and incubated at 37°C overnight. Single colonies were picked the next day and plasmids were isolated from these clones. Isolated plasmids were double-digested with *Eco*RI and *Nde*I to verify the cloning of the *GUN4* gene. The *GUN4-pDBle* construct from the selected clone was sequenced by the UC, Berkeley DNA Sequencing Facility (Berkeley, CA). Chromas Lite ( http://technelysium.com.au/) and BLAST were used to analyze DNA sequences.

**Table 5.** List of primers used for cloning and complement testing. These primers were used in the experiments that generated the data in Figure 8 and Figure 11 and were also used for *GUN4* gene amplification for cloning.

Primer name	Sequence of primer	Purpose
GUN4F1	5´-GGAATTCCATATGCTGGCCCAAACACACACT-3´	Amplification of *GUN4* for cloning
GUN4R1	5´-CCGGAATTCTTAGAACAGCGACTGTGTCCGCC-3´	Amplification of *GUN4* for cloning and for complement testing
PsaDF1	5´-CCACTGCTACTCACAACAAGCCCA-3´	Complement testing

### Generation and screening of *gun4* complements

Complementation of the *gun4-II* was performed utilizing the glass bead transformation technique described by Kindle *et al.* 1989 ^34^. 2 µg of the linearized *GUN4-pDBle* was used to complement *6F14*. Transformed cells were plated onto fresh TAP plates containing 15 µg/ml zeocin (Z) and placed in the dark at 25°C. Single colonies were picked and transferred onto fresh TAP+Z plates using a numbered grid template for screening of potential *gun4* complements. Screening of *gun4-II* complements was done by monitoring the Chl content and growth of complement strains either on TAP or HS plates under medium light (300 µmol photons m ^-2^ s ^-1^) in the presence or absence of antibiotics zeocin and paromomycin.

### Cellular protein analysis

*Chlamydomonas* cells from different strains grown in TAP in the dim light were harvested, washed twice with fresh medium and resuspended in TEN buffer (10 mM Tris-HCl, 10 mM EDTA and 150 mM NaCl; pH 8). Gel lanes were loaded with an equal amount of Chl (4 µg Chl). Resuspended cell suspension was mixed in a 1:1 ratio with the sample solubilization buffer SDS-urea buffer (150 mM Tris-HCl, pH 6.8; 7% w/v SDS; 10% w/v glycerol; 2 M urea; bromophenol blue and 10% β-mercaptoethanol) and were incubated at room temperature for about thirty minutes, with intermittent vortexing. The sample solubilization buffer was prepared according to the protocol of Smith *et al.* (1990) ^36^ using reagents from Fisher (Pittsburgh, PA). After incubation, the solubilized protein samples were vortexed and spun at a maximum speed of 20,000 *g* in a 1.5 ml eppendorf tube (USA Scientific, Ocala, FL) for five minutes at 4°C. The soluble fraction was loaded on a “any kD™ Mini-PROTEAN ^®^ TGX™ Precast Gel” (BioRad, Hercules, CA) and SDS-PAGE analysis was performed according to Laemmli (1970) ^37^ using a Page Ruler prestained molecular weight protein ladder (Fermentas, Glen Burnie, Maryland) at a constant current of 80 V for 2 hours. Gels were stained with colloidal Coomassie Gel Code blue stain reagent (Thermo Fisher Scientific, Rockford, IL) for protein visualization.

### Western analysis

Electrophoretic transfer of the SDS-PAGE resolved proteins onto an Immobilon P–PVDF membrane (Millipore, Billerica, MA) was carried out for 2 hours at a constant current of 400 mA in the transfer buffer (25 mM Tris, 192 mM glycine and 20% methanol). The GUN4 polyclonal antibody was raised in rabbit against the full length *Chlamydomonas* GUN4 mature protein that lacks the first 45 amino acids corresponding to the predicted chloroplast transit peptide ^31^. This antibody was generated by Dr. Roberto Bassi’s laboratory (University of Verona, Italy) and was provided to us by Dr. Krishna Niyogi (UC, Berkeley). GUN4 primary antibodies were diluted to a ratio of 1:1000 before being used as a primary probe. The secondary antibodies used for Western blotting were conjugated to horseradish peroxidase (Pierce protein research product, Thermo Fisher Scientific, Rockford, IL) and diluted to a ratio of 1:20,000 with the antibody buffer. Western blots were developed by using the Supersignal West Pico chemiluminescent substrate kit (Pierce protein research product, Thermo Fisher Scientific, Rockford, IL).

### Cell counts and chlorophyll extraction

Cell density (number of cells per ml of the culture) was calculated by counting the cells using a Neubauer ultraplane hemacytometer (Hausser Scientific, Horsham, PA). Pigments from intact cells were extracted in 80% acetone and cell debris was removed by centrifugation at 10,000 *g* for 5 minutes. The absorbance of the supernatant was measured with a Beckman Coulter DU 730 Life Science UV/Vis spectrophotometer (Brea, CA). Chl *a* and *b* concentrations were determined by Arnon (1949) ^38^ equations, with corrections as described by Melis *et al.* (1987) ^39^.

## Results

### Generation and identification of the mutant *6F14*

**Figure 3.**
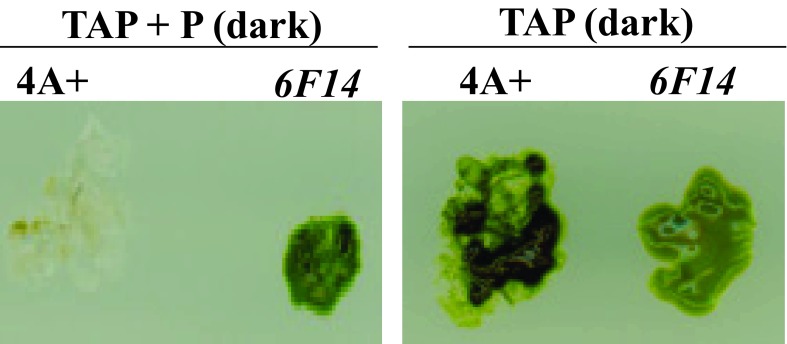
Identification of *6F14*. This figure shows the phenotypic difference of *6F14* compared to the parental strain, 4A+ on heterotrophic agar media (TAP) plates under two different growth conditions: dark + paromomycin (P) and dark.

Mutant *6F14* was generated by random insertional mutagenesis of the *C. reinhardtii* wild type strain 4A+ (137c genetic background). *6F14* was identified as a slightly Chl deficient paromomycin resistant mutant on TAP+P plate in the dark ( Figure 3).

### Growth analyses of *6F14*

**Figure 4.**
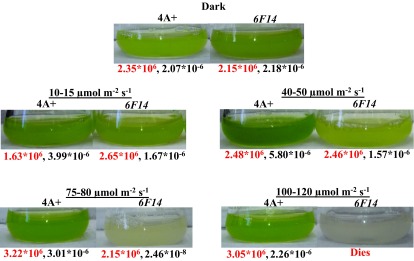
Heterotrophic and mixotrophic growth of *6F14* and the wild-type in TAP media. Dark adapted cells of *6F14* and 4A+ were shifted to different light intensities in this experiment. Light conditions and strains are labeled above the culture flasks. The cell density (cells/ml) and nmol chlorophyll (Chl) per cell are shown below the culture flasks in red and black numbers, respectively. For each light condition, experiments were performed on three biological replicates of each strain. Statistical error (±SD) was ≤ 10%.

**Figure 5.**
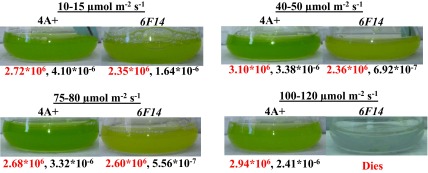
Photo-autotrophic growth of *6F14* and wild-type in HS media. Dark adapted cells of *6F14* and 4A+ were shifted to different light intensities in this experiment. The mean cell density (cells/ml) and the Chlorophyll (Chl) content (nmol Chl per cell) are shown below the culture flasks in red and black numbers, respectively. For each light condition, experiments were performed on three biological replicates of each strain. Statistical error (±SD) was ≤ 10%.

Growth analyses in heterotrophic and photo-autotrophic liquid media revealed that *6F14* is light sensitive and shows progressive photo-bleaching with increase in light intensities ( Figure 4 and Figure 5). In mixotrophic conditions under 10–15 µmol photons m ^-2^ s ^-1^, *6F14* possesses 58% less Chl/cell than 4A+. At 40–50 µmol photons m ^-2^ s ^-1^, *6F14* has 72% less Chl/cell than the wild type. At 75–80 µmol photons m ^-2^ s ^-1^, *6F14* possesses 99% less Chl/cell than the wild type. At 75–80 µmol photons m ^-2^ s ^-1^, *6F14* starts to photo-bleach and turns yellow; it dies at light intensities 100–120 µmol photons m ^-2^ s ^-1^ in TAP ( Figure 4).

Figure 5 shows photo-autotrophic cultures of *6F14* and 4A+. *6F14* has the ability to grow photo-autotrophically in HS media in dim light (10–15 µmol of photons m ^-2^ s ^-1^). However, the mutant grows extremely slowly in comparison to the wild type. When grown at 10–15 µmol photons m ^-2^ s ^-1^ in HS media, *6F14* possesses 60% less Chl/cell than the wild type. At 40–50 µmol photons m ^-2^ s ^-1^ in HS media, *6F14* has 79% less Chl/cell than 4A+, and at 75–80 µmol photons m ^-2^ s ^-1^ in HS media, *6F14* possesses 83% less Chl/cell than the wild type. At 100–120 µmol photons m ^-2^ s ^-1^ in HS media, *6F14* fails to survive ( Figure 5).

**Figure S1.**
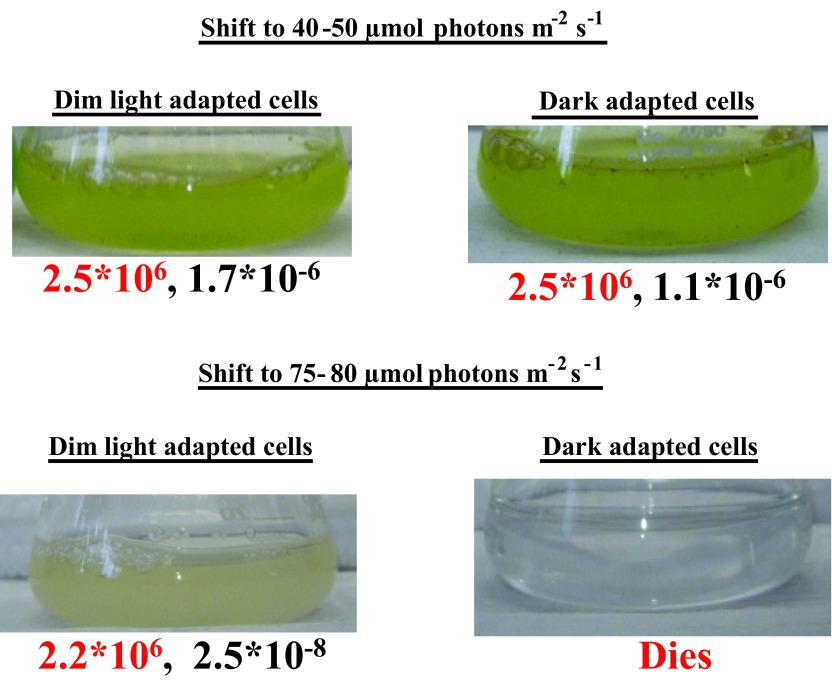
Effect of light shift on the growth of *6F14* and wild type. *6F14* was adapted to dim light (10–15 µmol photons m ^-2^ s ^-1^) or dark for one week in TAP media. Dark and dim light adapted cultures were then shifted to 40–50 or 75–80 µmol photons m ^-2^ s ^-1^. The mean cell density (cells/ml) and the Chlorophyll (Chl) content (nmol Chl per cell) are shown below the culture flasks in red and black numbers, respectively. For each light condition, experiments were performed on three biological replicates of *6F14*. Statistical error (±SD) was ≤ 10%. The average Chl content in the dim light and dark adapted *6F14* was 1.7 × 10 ^-6^ and 2.18 × 10 ^-6^ nmol/cell.

Figure S1 demonstrates that when dim light adapted *6F14* was shifted to 40–50 µmol photons m ^-2^ s ^-1^ there was no significant change in Chl/cell content ( Figure 4). Dark adapted *6F14* showed a 50% reduction in Chl/cell when moved to 40–50 µmol photons m ^-2^ s ^-1^. When dim light adapted *6F14* was shifted to 75–80 µmol photons m ^-2^ s ^-1^, it showed a 98% reduction in Chl/cell while the dark adapted *6F14* failed to survive under 75–80 µmol photons m ^-2^ s ^-1^. Taken together, the results shown in Figure 4 and Figure S1 show that dark adapted *6F14* is more sensitive to the magnitude of light intensity changes in the environment than the dim light adapted *6F14* ( Figure S1).

### Molecular characterization of the mutation in *6F14*

**Figure S2.**
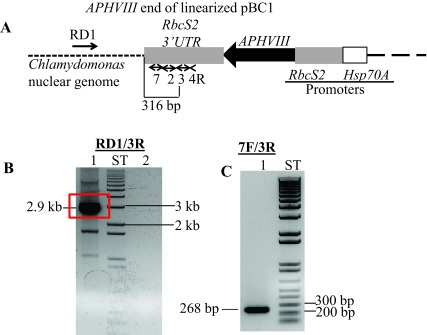
Locating the *APHVIII* flanking genomic sequence in *6F14*. ( **A**) A diagram showing a truncated pBC1 illustrating the *APHVIII* end of the linearized pBC1 vector. Primers used for PCR and DNA sequencing are shown by numbered black arrows. Thermal Asymmetric InterLaced1 (TAIL1) PCR was performed using primers 4R and RD1 (a random degenerate primer). ( **B**) TAIL2 PCR was performed using primers 3R and RD1. In lane 1, 10-fold diluted TAIL1 PCR product was used for TAIL2 PCR; Lane 2 is a zero DNA control lane. The 2.9 kb TAIL2 PCR product used for DNA sequencing is highlighted in the red box. Initial DNA sequencing was performed using vector specific primers 2R and 3R ( Table 1). ( **C**) Gel purified DNA product (2.9 kb) from TAIL2 PCR was used to verify if the product is specific to the *APHVIII* gene. PCR primer names are labeled on the top of the gel. PCR product size is labeled. F and R stand for forward and reverse primers, respectively. All primer sequences are shown in Table 1. ST stands for 1 kb plus ladder (Invitrogen, Carlsbad, CA). DNA samples were run on a 1% agarose gel.

**Figure S3.**
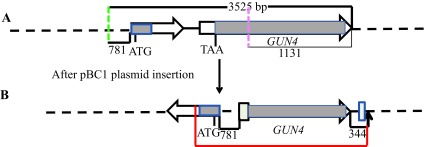
A schematic of the genetic rearrangement in *6F14* based on TAIL PCR analyses. ( **A**) A schematic genomic map showing a 3525 bp genomic DNA region spanning the *GUN4* locus. The number at the bottom of the map denotes distance between respective points on the genomic DNA. The two *GUN4* exons are represented by white block arrows. Grey boxes in *GUN4* denote UnTranslated Regions (UTRs). The two break points are shown by the dashed pink and green lines. ( **B**) A schematic diagram showing the rearrangement of the *GUN4* locus after the insertion of the plasmid. The big and the small tan boxes, denote addition of 45 and 29 bp, respectively. The genomic DNA sequence obtained by sequencing the 2.9 kb Thermal Asymmetric InterLaced2 (TAIL2) PCR product is highlighted in red. The bold black small arrow indicates insertion point of the pBC1 plasmid. DNA sequencing was performed using *GUN4* specific primers 2R, 7F and 7R, 12R and 14R. F and R stand for forward and reverse primers, respectively. Primer sequences are shown in Table 2.

The linearized pBC1 plasmid was used to generate *6F14* ( Figure 2). To find the insertion of the *APHVIII* end of the plasmid in *6F14*, TAIL PCR method was employed. Figure S2A shows the position of the vector specific TAIL PCR primers and also shows the arbitrary position of the random degenerate primer. A 2.9 kb DNA product from TAIL2 PCR was purified from the agarose gel ( Figure S2B, Table 1). This purified DNA product was used for further PCR using internal primers specific to the 3´ UnTranslated Region (UTR) of the *APHVIII* gene. The PCR results confirmed that the 2.9 kb DNA product contains the 3´ UTR of the *APHVIII* gene ( Figure S2C). Sequencing of the 2.9 kb TAIL2 PCR product revealed that the *APHVIII* end of the plasmid has been inserted 344 bp away from the *GUN4* gene (Cre05.g246800) on chromosome 5. The *GUN4* locus was cleaved at least at two places ( Figure S3). The first cleavage was about 781 bp away from the 5′ end of the *GUN4* gene and the second cleavage was 1131 bp away from the 3´ end of the *GUN4* gene. These cleavages were followed by the inversion of the cleaved genomic DNA which then ligated to the 3´ UTR of the *GUN4* gene ( Figure S3). Plasmid insertion also led to an addition of 29 bp at the *APHVIII* end of the plasmid. An addition of 45 bp was found at the breakage point in the 3´ UTR of the *GUN4* gene ( Figure S3).

**Figure 6.**
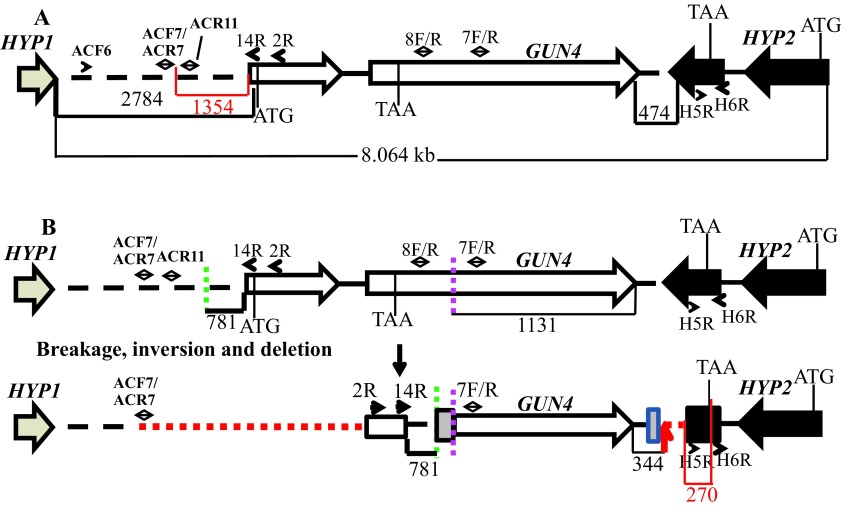
A schematic of the genetic rearrangement in *6F14*. ( **A**) A schematic genomic map showing an 8.064 kb genomic DNA region spanning the *GUN4* locus on chromosome 5. The numbers at the bottom of the map denote distances between respective points on the genomic DNA. The red highlighted region and number show the distance between primer ACF7 and the start of the *GUN4* gene and the distance between the primer H5F and the end of the *HYP2* gene, respectively. The two *GUN4* exons are represented by white block arrows. The tan arrow and the black block arrow, denotes a part of *HYP1* 3´ UTR and *HYP2* gene, respectively. ( **B**) An updated schematic diagram showing the rearrangement of the *GUN4* locus based on PCR analyses and DNA sequencing. Two break points in the genome are denoted by green and pink dashed lines. The big and the small grey boxes, denote addition of 45 and 29 bp, respectively. The small black arrows denote primers that were used for genomic PCRs in Figure S4 and Figure S5. Red dashed lines denote possible deletions. The small red arrow indicates the point of insertion of the pBC1 plasmid. The black numbers at the bottom of the map denote distances between respective points on the genomic DNA. The red highlighted region and the corresponding number show the distance between the end of the primer H5F and the stop codon of the *HYP2* gene.

**Figure S4.**
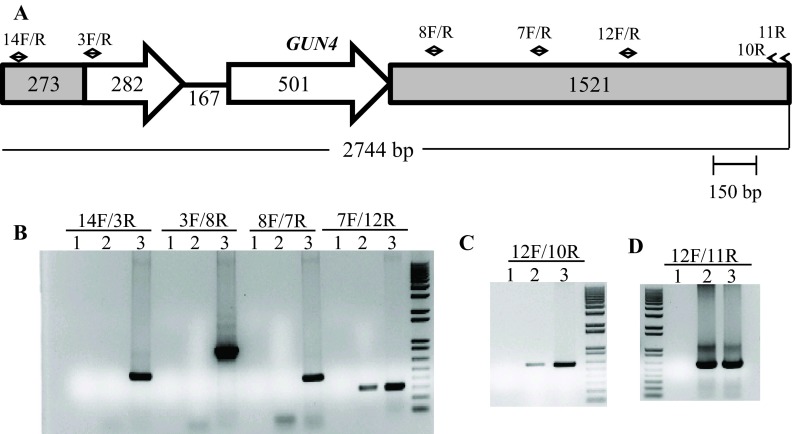
Genomic DNA analysis of *6F14* and 4A+. ( **A**) A schematic of the *GUN4* gene. Small black arrows denote *GUN4* specific primers. The two *GUN4* exons are represented by white block arrows. Grey boxes in *GUN4* denote UnTranslated Regions (UTRs). ( **B**), ( **C**) and ( **D**) DNA gels showing DNA products obtained from genomic DNA PCR using *GUN4* specific primers. Lanes 1, 2 and 3 are the zero DNA controls, *6F14* and 4A+, respectively. PCR primer names are labeled on the top of the gel. 14F/3R gives a 517 bp product while 3F/8R gives a 942 bp product. 8F/7R gives a 449 bp product. 7F/12R gives a 313 bp product. 12F/10R gives a 606 bp product and 12F/11R gives a 623 bp product. F and R stand for forward and reverse primers, respectively. Primer sequences are shown in Table 2.

**Figure S5.**
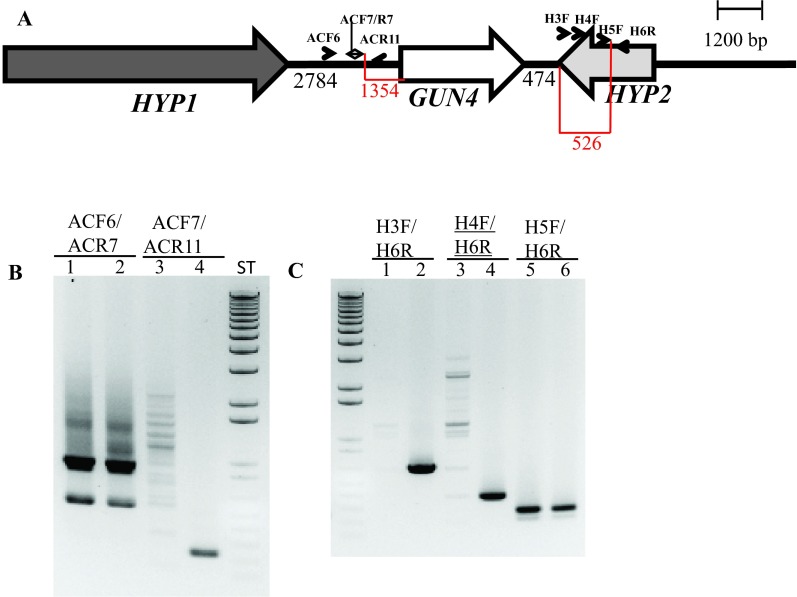
PCR using primers specific to the upstream region of *GUN4* and the 3´ UTR of the *HYP2* gene. ( **A**) A schematic diagram showing *GUN4* (white arrow) and its two neighboring genes *HYP1* (black arrow) and *HYP2* (grey arrow). Black numbers at the bottom denote distances between respective genes. The two red highlighted regions and corresponding numbers show the distance between primer ACF7 and the start of the *GUN4* gene and the distance between the primer H5F and the end of the *HYP2* gene, respectively. Primers used for PCR are labeled. ( **B**) A DNA gel showing the genomic DNA amplified using *GUN4* upstream region specific primers. Lanes 1, 3 and 5: *6F14*; Lanes 2, 4 and 6: 4A+. ( **C**) A DNA gel showing the genomic DNA amplified using primers spanning the 3´ UTR region of *HYP2* gene. Lanes 1, 3 and 5: *6F14*; Lanes 2, 4 and 6: 4A+. Product size of ACF6/ACR7: 937 bp (this primer set also produces a nonspecific 550 bp product); product size of ACF7/ACR11: 244 bp; product size of H3F/H6R: 667 bp; product size of H4F/H6R: 391 bp; product size of H5F/H6R: 278 bp. F and R stand for forward and reverse primers, respectively. Primer sequences are shown in Table 3.

Further genomic DNA PCR analyses with *GUN4* specific primers confirmed that the 3´ part of the *GUN4* first exon and the 5′ part of the *GUN4* second exon were deleted or displaced ( Figure S4). We also used primers specific to the genomic region upstream of the *GUN4* gene and primers specific to the 3´ UTR of a hypothetical gene, *HYP2*, (g5195) located downstream of *GUN4* to see the extent of deletion on either side of the *GUN4* gene. Our PCR analyses show that a 1.354 kb genomic DNA region, located upstream of *GUN4* was deleted/displaced. Additionally, there is a deletion of approximately 526 bp in the 3´ UTR of the downstream *HYP2* gene ( Figure S5 and Figure 6). Taken together the data show that plasmid insertion in the *6F14* genome has rearranged the *GUN4* locus and has affected a part of the 3´ UTR of the *HYP2* gene. We do not yet know the exact location of the pUC ori end of the plasmid in the *6F14* genome ( Figure 2).

### Checking for the absence/presence of the transcript of the *GUN4* and three neighboring genes of *GUN4*

**Figure 7.**
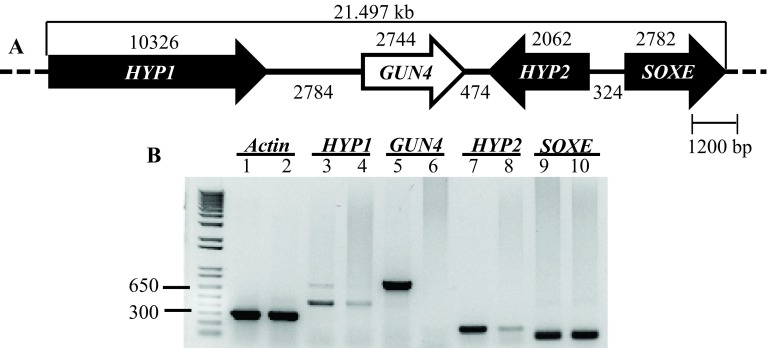
Transcript analyses of *GUN4* and its neighboring genes. ( **A**) A schematic map of a 21.497 kb genomic region spanning the *GUN4* locus on chromosome 5. *HYP1* and *HYP2* are genes located upstream and downstream of the *GUN4* gene, respectively coding for hypothetical proteins. Sulfocyanin ( *SOXE*) codes for a blue copper protein. The top black number denotes size of a gene (bp) while the bottom black number denotes distance between genes (bp). ( **B**) Semi-quantitative RT-PCR results. Lanes: 1, 3, 5, 7, 9 denote 4A+ cDNA products. Lanes 2, 4, 6, 8, 10 denote *gun4* cDNA products. Primer sequences are shown in Table 4. All primers span an intron. *Actin* was used as a control. *Actin* genomic product size: 527 bp; *Actin* cDNA product size: 305 bp. *HYP1* genomic product size 726 bp; *HYP1* cDNA product size: 459 bp. *GUN4* genomic product size: 942 bp; *GUN4* cDNA product size 775 bp. *HYP2* genomic product size: 797 bp; *HYP2* cDNA product size: 184bp. *SOXE* genomic product size: 517 bp; *SOXE* cDNA product size: 119 bp.

Transcript levels of *GUN4* and the neighboring genes ( *HYP1* [Cre05.g246750]; *HYP2* [g5195] and *SOXE* [Cre05.g246900]) were checked using semi-quantitative RT-PCR using *GUN4*, *HYP1*, *HYP2* and *SOXE* specific primers, respectively ( Figure 7). Reduced levels of *HYP1* and *HYP2* transcripts were observed in *6F14* compared to that in the wild type ( Figure 7). *GUN4* transcript is missing in *6F14* as expected ( Figure 7). The transcript level of *SOXE,* the second gene downstream of *GUN4*, was not affected. Cre05.g246750 and g5195 are genes in the *Chlamydomonas* database coding for hypothetical proteins. We have named these genes as *HYP1* and *HYP2* arbitrarily for our study. The *SOXE* gene codes for sulfocyanin, a blue copper protein. Readers are requested to identify *GUN4* and its neighboring genes by the gene locus number (Cre or the g number) in the Phytozome database.

### Complementation of *gun4-II*

**Figure 8.**
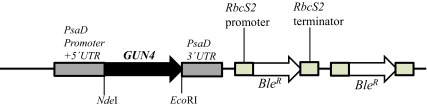
A schematic figure of the pDBle vector used for complementation of *gun4-II*. *Nde*I/ *Eco*RI double digested *GUN4* gene (956 bp) was cloned into the *Nde*I/ *Eco*RI double digested pDBle plasmid. Primers used for amplification of the *GUN4* gene are shown in Table 5. *GUN4* expression is driven by the constitutive *PsaD* promoter. *Nde*I and *Eco*RI restriction sites are labeled. pDBle contains two copies of *Ble ^R^* genes driven by the Rubisco ( *RbcS2*) promoter. The size of the *GUN4*- *pDBle* construct is 7653 bp. The black arrow and the white arrow, denotes *GUN4* and *Ble ^R^* genes, respectively. Grey and tan boxes denote UnTranslated Regions (UTRs).

**Figure 9.**
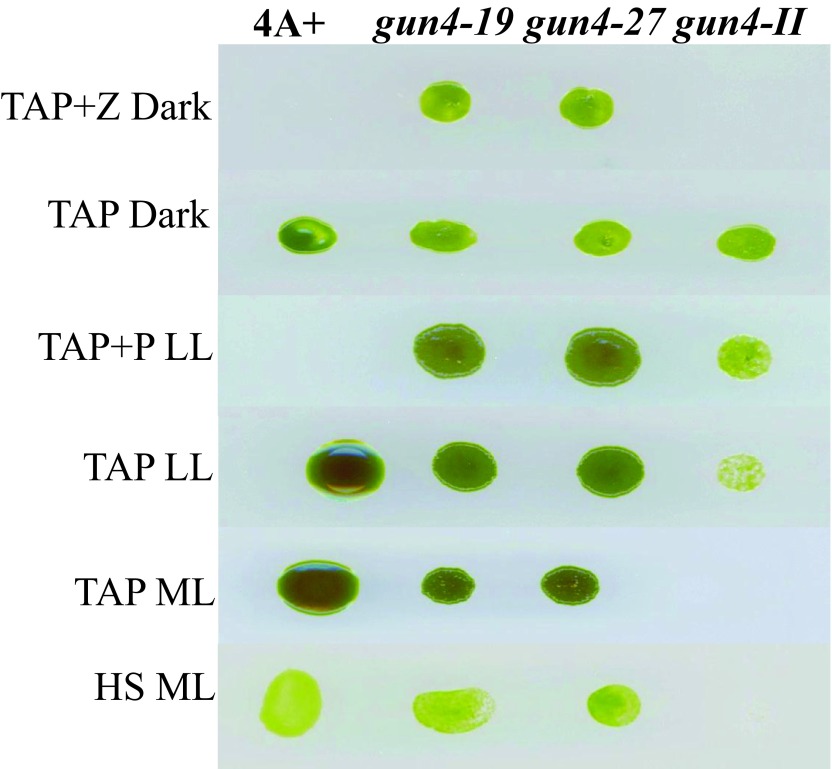
Growth phenotype analysis of *gun4-II* complements. *gun4-19* and *gun4-27* complements, *gun4-II* and 4A+ were grown for a week under six different growth conditions: TAP + Z (zeocin) in the dark, TAP in the dark, TAP + P (paromomycin) low light (LL; 50 µmol photons m ^-2^ s ^-1^), TAP LL, TAP medium light (ML; 300 µmol photons m ^-2^ s ^-1^) and HS ML.

We will be referring to *6F14* as *gun4-II* from here onward. As *gun4-II* specifically lacks a functional *GUN4* gene, we cloned the *GUN4* gene in the pDBle vector to transform *6F14* ( Figure 8, Table 5). The trans *GUN4* expression is driven by the constitutive *PsaD* promoter in the *GUN4-pDBle* construct. pDBle has two *Ble* genes that confer resistance to the antibiotic zeocin. Figure 9 shows growth phenotypes of two *gun4-II* complements ( *gun4-19* and *gun4-27*), *6F14* and 4A+. *gun4-II* complements are not light sensitive and are able to grow and photosynthesize under medium light intensities (300 µmol photons m ^-2^ s ^-1^) without photo-bleaching ( Figure 9). As *gun4-II* complements harbor the *Ble* gene (from the pDBle vector) and *APHVIII* gene (derived from the parental strain *gun4-II*), they can grow both on zeocin and paromomycin media plates unlike *gun4-II* and 4A+ ( Figure 9).

**Figure 10.**
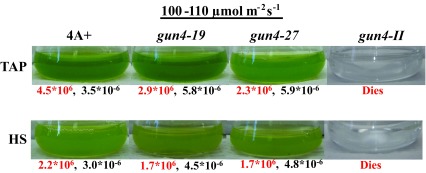
Mixotrophic and photo-autotrophic growth of *gun4-II* complements. Light intensity is labeled above the culture flask. Growth media is labeled to the left of the culture flask. The mean cell density (cells/ml) and the Chlorophyll (Chl) content (nmol Chl per cell) are shown below the culture flasks in red and black numbers, respectively. For each light condition and growth condition, experiments were performed on three biological replicates of each strain. Statistical error (±SD) was ≤ 10%.

**Figure 11.**
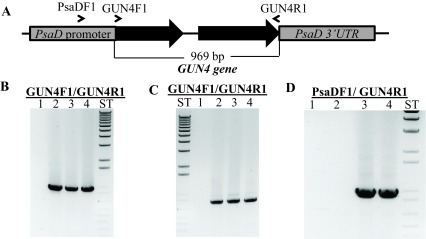
Molecular analyses of *gun4-II* complements. ( **A**) A schematic diagram of the *GUN4*-pDBle construct. Primers used for PCR are shown on the map. ( **B**) Genomic DNA PCR analyses with *GUN4* cloning primers (product size: 969 bp). Lane 1: *gun4-II*; Lane 2: 4A+; Lane 3: *gun4-19*; Lane 4: *gun4-27*. ( **C**) RT-PCR analyses with *GUN4* cloning primers (product size: 802 bp). Lane 1: *gun4*; Lane 2: 4A+; Lane 3: *gun4-19*; Lane 4: *gun4-27*. ( **D**) Genomic PCR analyses using a *PsaD* 5′ UTR specific forward primer with a *GUN4* cloning reverse primer (product size: 976 bp). Lane 1: *gun4-II*; Lane 2: 4A+; Lane 3: *gun4-19*; Lane 4: *gun4-27*. Primer sequences are shown in Table 5.

**Figure 12.**
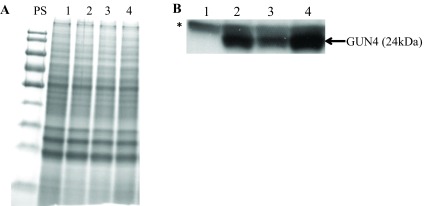
SDS-PAGE and Western analyses. ( **A**) A stained protein gel. Lanes 1, 2, 3 and 4 represent *gun4-II, gun4-19*, *4A*+ and *gun4-27*, respectively. PS denotes prestained molecular weight protein ladder. Total cell extract of different strains were loaded on equal Chlorophyll (Chl) basis (4 µg of Chl). ( **B**) Western analyses using a GUN4 antibody generated against the *Chlamydomonas* mature full length GUN4 protein. Lanes 1, 2, 3 and 4 represent *gun4-II, gun4-19, 4A*+ and *gun4-27*, respectively. GUN4 (24 kDa) protein detected by the antibody is labeled. * denotes a 25 kDa protein detected non-specifically by the GUN4 antibody.

Chl analyses show that under heterotrophic conditions both *gun4-II* complements have 65–68% more Chl than that of the wild type cells ( Figure 10). Under photo-autotrophic conditions *gun4-II* complement cells possess 50–60% more Chl than that of the wild type cells ( Figure 10). Figure 11A shows a schematic figure of the trans *GUN4* gene used for complementation. PCR analyses using the genomic DNA show that the *gun4-II* complements possess the functional trans *GUN4* gene ( Figure 11B, 11C and 11D). Figure 12A shows a stained protein gel that was loaded on equal Chl basis. Western analyses of the two *gun4-II* complements with a *Chlamydomonas* GUN4 specific antibody show that the GUN4 protein is absent in the *gun4-II* mutant but present in the *gun4* complements ( Figure 12B). Western analyses also show that the two *gun4-II* complements have higher levels of the GUN4 protein compared to that of the wild type ( Figure 12B).

Spectrophotometric chlorophyll analyses in 6F14, 4A+ and gun4 complements. The cultures were tested under a range of light intensites. The light intensity shown under the light column denotes the lower limit of the light intensity range in µmol photons m-2s-1. 10 denotes a range of 10-15 µmol photons m-2s-1; 40 denotes a range of 40-50 µmol photons m-2s-1; 75 denotes a range of 75-80 µmol photons m-2s-1 and 100 denotes a range of 100-110 µmol photons m-2s-1. 6F14 does not survive under 100-110 µmol photons m-2s-1. The cultures were tested shifted to a range of light intensity. The light intensity shown under the light column denotes the lower limit of the light intensity range in µmol photons m-2s-1. 40 denotes a range of 40-50 µmol photons m-2s-1 and 75 denotes a range of 75-80 µmol photons m-2s-1. Dark adapted 6F14 shifted to 75-80 µmol photons m-2s-1 did not survive. The cultures were tested under a range of light intensity. The light intensity shown under the light column denotes the lower limit of the light intensity range in µmol photons m-2s-1.100 means 100-110 µmol photons m-2s-1. 6F14 fails to survive under 100-110 µmol photons m-2s-1 in TAP and HS media.

## Discussion

Plastid development and gene expressions are largely under nuclear “anterograde” control ^40^. Additionally, chloroplast functional and developmental states can regulate expression of nuclear genes encoding chloroplast localized proteins via retrograde signaling ^40^. The first evidence for the involvement of Chl biosynthetic precursors in retrograde signaling came from the work in *Chlamydomonas* ^41^. In *Arabidopsis* MgPPIX was hypothesized to be a retrograde signal from the chloroplast to the nucleus on the basis of data obtained with mutants that are defective in the norflurazon (NF) induced down-regulation of transcription of light harvesting complex protein B (LHCB) [gun (genomes uncoupled) phenotype] ^40, 42^. Six *gun* mutants are known; five of which directly influence tetrapyrrole biosynthesis *(gun2-gun6)* ^43, 44^. The *gun4* mutation is localized to a porphyrin binding protein GUN4. GUN4 enhances the sensitivity of MgChel to Mg ^2+^ at physiologically low Mg ^2+^ concentration ^31, 45^. Cyanobacterial and higher plant GUN4 directly interacts with the CHLH subunit of MgChel and binds PPIX and MgPPIX, the substrate and the reaction product of the MgChel ^30, 46– 50^. Although GUN4 is not an essential component of the MgChel complex, the presence of GUN4 markedly improves the enzyme activity *in vitro* by increasing the apparent substrate-binding capacity of CHLH for PPIX, particularly under low Mg ^2+^ concentrations ^45, 51, 52^. It is proposed that GUN4 upon porphyrin binding, stabilizes interactions between the catalytic subunit of MgChel and the chloroplast membranes, the site of Chl biosynthesis ^46, 47^. This enables MgChel to interact with enzyme complexes involved in the further downstream steps in the pathway ^46, 47^. Apart from its role in substrate channeling into the Chl synthesizing branch of tetrapyrrole biosynthesis, GUN4 has also been implicated in providing photo-protection under increasing light intensities ^30, 46, 47^. The porphyrin binding property of GUN4 has been implicated in ROS attenuation but conclusive experimental support is lacking ^47^. In higher plants, GUN4 has been implicated as an essential component in a post-translational feedback regulation mechanism that modulates ALA biosynthesis in response to enzymatic activities of the Mg branch of tetrapyrrole biosynthesis as well as to the accumulating Mg porphyrin levels ^30^ ( Figure 1).

*6F14* is the second *gun4* mutant ( *gun4-II*) to be identified in *C. reinhardtii*. The first *C. reinhardtii gun4* mutant was identified and characterized in 2012 by Formighieri *et al.* ^31^. In this *gun4* mutant, 184 bp of the second exon of the *GUN4* gene is deleted. In *gun4-II* the plasmid insertion outside the *GUN4* gene has caused a genetic rearrangement of the *GUN4* gene that prevented gene expression ( Figure 6). Transcripts of *GUN4* and the neighboring genes of *GUN4* in *gun4-II* were checked by performing semi-quantitative reverse transcription PCR. In *gun4-II*, the transcript level of the first downstream hypothetical ( *HYP2*) gene was lower than that in the wild type ( Figure 7). The plasmid insertion in *gun4-II* has led to a deletion of part of the 3´ UTR region of the *HYP2* gene (270 bp away from the stop codon of the coding region of *HYP2;* Figure 6). The 3´ UTR is usually responsible for the stability of the transcript. Hence the nature of the deletion in *HYP2* explains the decrease in transcript levels of *HYP2*. *GUN4* and the upstream gene, *HYP1*, are separated from each other by 2.784 kb ( Figure S5). There is a possible deletion/genetic rearrangement in the 5′ genomic region upstream of the *GUN4* which does not extend into the *HYP1* gene ( Figure S5 and Figure 6). Although the transcription of *HYP1* was not hampered, the *HYP1* transcript level is lower in our *gun4* compared to that in the wild type ( Figure 7). Based on the RT-PCR analyses, it is speculated there might be some uncharacterized downstream regulatory sequences present in the 2.784 kb region that might regulate *HYP1* transcription. In future, quantitative real time-PCR experiments can be used to accurately quantify transcript levels of *HYP1* and *HYP2* in *gun4-II*.

The photosensitive phenotype of our *gun4-II* mutant resembles that of the earlier identified *Chlamydomonas gun4* mutant which we will refer from here onward, as *gun4-I*. Over-accumulation of photo-excitable PPIX leads to photo-oxidative damage to the cells in presence of light and oxygen ^4, 26, 53^. The light sensitivity of *gun4-II* is most probably due to an over-accumulation of the PPIX which occurs due to the inactivity of MgChel enzyme as has been shown by Formighieri *et al.* (2012) ^31^ in the *gun4-I* mutant. Future HPLC (High Performance Liquid Chromatography) analyses of steady state tetrapyrrole intermediates in *gun4-II* will confirm this hypothesis. Formighieri *et al.* (2012) ^31^ explored four light conditions (dark, 6-, 50-, and 500 µmol photons m ^-2^ s ^-1^) and showed that the *gun4-I Chlamydomonas* mutant dies under high light (500 µmol photons m ^-2^ s ^-1^). These researchers did not explore or clarify the maximum light irradiance condition that can be tolerated by the *Chlamydomonas gun4-I* mutant in heterotrophic and photosynthetic growth conditions. In this study, we found that *gun4-II* photo-bleached at 75–80 µmol photons m ^-2^ s ^-1^ and could not tolerate light intensity above 100 µmol photons m ^-2^ s ^-1^ ( Figure 4 and Figure 5). The earlier identified *C. reinhardtii gun4-I* mutant is able to grow in continuous light slightly better than in photoperiodic shifts ^31^. In *Arabidopsis*, the *gun4* mutant is seen to exhibit significant improved growth in continuous light compared to periodic shifts in light ^30^. In this study, *gun4-II* and the wild type were adapted to dark or dim light and then shifted to two different light irradiances (40–50 µmol photons m ^-2^ s ^-1^ and 75–80 µmol photons m ^-2^ s ^-1^). Cultures exposed to light shifts showed a significant reduction in the Chl content than those grown under a constant light intensity ( Figure 4 and Figure S1). Additionally, dark adapted *gun4-II* showed a significant reduction in the Chl content compared to the dim light adapted *gun4-II*, when cells were shifted to similar light intensities ( Figure 6). These results show that *gun4-II* is very sensitive to the magnitude of light intensity fluctuations in the environment unlike the earlier reported *Chlamydomonas gun4-I* mutant ^31^. Our light shift experimental results support the findings in cyanobacterial and *Arabidopsis gun4* mutants ^30, 48– 50^.

By spectrophotometric analysis we have shown that in the dark *gun4-II* possesses almost similar Chl content like that in the wild type ( Figure 4). This phenotype is very different from that of *gun4-I*, which possesses 50% of the wild type level of Chl/cell ^31^ in the dark. Variation in Chl/cell in the dark between the two *C. reinhardtii gun4* mutants could possibly be due to a variation of the parental strain’s ability to synthesize Chl in the dark. The parental strain used by Formighieri *et al.* (2012) ^31^ was *cw15mt-*. However, the 50% decrease in Chl seen in the the *gun4-I* mutant was determined through HPLC analyses. Hence the discrepancy in Chl content in the two *gun4* mutants could be due to the sensitivity of the HPLC method compared to that of the spectrophotometric method used for Chl assays.

Steady state tetrapyrrole analyses by HPLC can be performed to check the various tetrapyrrole intermediate accumulation in *gun4-II* under different light conditions. Measurements of ALA biosynthesis rate in gun4-II can show if GUN4 also regulates earlier steps in the tetrapyrrole biosynthetic pathway, as suggested by some researchers ^48^.

Interestingly, it has been shown by Formighieri *et al.* (2012) ^31^ that *Chlamydomonas gun4-I* complements expressing more GUN4 protein grow better under high light and that there is no correlation between the accumulation of PPIX and the ability to grow better under high light ^31^. However these *gun4-I* complements were not over-expressers of the GUN4 protein compared to the wild type strain *cw15*, used in their experiments ^31^. Our two *gun4-II* complements ( *gun4-19* and *gun4-27*) are over-expressing the GUN4 protein compared to the wild type strain 4A+ in the dim light ( Figure 12B). These two *gun4-II* complements open up new avenues to test if GUN4 has a distinct photo-protective role that is independent from the PPIX-induced GUN4 photo-protective role proposed by several researchers ^46, 47^. Comparative growth studies, quantitative measurements of *GUN4* transcripts by Real Time PCR, GUN4 protein levels by Western analyses and PPIX content by HPLC analyses of the high light-(500 µmol photons m ^-2^ s ^-1^) and dim light-(15–20 µmol photons m ^-2^ s ^-1^) adapted *gun4-II* complements and the wild type strain will help to confirm if GUN4 has a distinct photo-protective role that is independent of tetrapyrrole metabolism.

Taken together our work reconfirms the results of other researchers who have studied GUN4 in other photosynthetic organisms ^30, 31, 46, 47, 49, 50^. Although loss of GUN4 caused a perturbation in Chl biosynthesis in *gun4-II* mutant, the effect is not as dramatic as it is in *Arabidopsis*, where the loss of GUN4 results in a nearly *albino* mutant ^51^. The earlier identified *Chlamydomonas gun4-I* mutant phenotypically resembles our *gun4-II* mutant ^31^. Therefore it seems that in *C. reinhardtii* Chl biosynthesis is less dependent on the GUN4 function. One explanation for this difference in the mutant phenotype could be that *Chlamydomonas* is capable of synthesizing Chl in the dark unlike the angiosperms. GUN4 interacts with PPIX and acts at the branch point in the tetrapyrrole biosynthetic pathway where PPIX is diverted to heme and Chl biosynthesis. Hence, although GUN4 has a conserved physiological role in all oxygenic photosynthetic organisms, it might have a different role in different evolutionary groups depending on the channelization of PPIX into the heme and Chl branch in the pathway.
