# Supplementary material for: Identification and molecular characterization of the second Chlamydomonas gun4 mutant, gun4-II
Source: F1000Res. 2013 Jul 29;2:142. Originally published 2013 Jun 19. [Version 2] doi: 10.12688/f1000research.2-142.v2 (PMC3931455; doi:10.12688/f1000research.2-142.v2)
Supplement: Peer review history

# Peer review: Referee response for version 2

**Published:** 2013-08-28

**Authors:** Kittisak Yokthongwattana

**Affiliations:** 1 Department of Biochemistry, Mahidol University, Bangkok, Thailand

Most of the comments I made to the original manuscript were addressed. I am now happy with it and I have no further critical comments.

I have read this submission. I believe that I have an appropriate level of expertise to confirm that it is of an acceptable scientific standard.

# Peer review: Referee response for version 1

**Published:** 2013-07-09

**Authors:** EonSeon Jin

**Affiliations:** 1 Department of Life Science, College of Natural Science, Hanyang University, Seoul, Korea, South

This manuscript presents data regarding phenotype of isolated mutants ( *6F14*), identification of the mutation locus in *6F14* and its complementation.The results revealed that *6F14* is defective in the GUN4 (genome uncoupled 4) gene which regulate MgChel activity. Therefore, the transformation of *6F14* with a GUN4 gene restored the wild type phenotype with over-expressing the GUN4 protein. The authors concluded that *6F14* is the second *gun4* mutant identified in *Chlamydomonas*. In that sense, the study of *6F14* ( *gun4 II*) is not novel. However, the authors have shown the difference of mutation locus of *gun4 I* (earlier identified) and *gun4 II* (in this study) and also the differences of light sensitivity and chlorophyll content which is very interesting. I do have some suggested revisions:

**1.** If the authors compare all physiological responses for two *gun4* mutants and their parental wild types in the same culture condition, it would explain more clearly the differences between the *gun4 II* and *gun4 I *mutants.  

**2.** Authors may need to propose the future experiment with this new *gun4* mutant ( *6F14*) in the discussion section of the article. **3.** I noticed that the introduction part of your abstract (up to 5 lines) is almost identical with your recent paper “ Identification and molecular characterization of a novel *Chlamydomonas reinhardtii* mutant defective in chlorophyll biosynthesis” paper. I would recommend you to revise the introduction part of this abstract.

I have read this submission. I believe that I have an appropriate level of expertise to confirm that it is of an acceptable scientific standard.

# Peer review: Referee response for version 1

**Published:** 2013-07-09

**Authors:** Kittisak Yokthongwattana

**Affiliations:** 1 Department of Biochemistry, Mahidol University, Bangkok, Thailand

This interesting paper by Mitra *et al.* presents results that show another allele of the *gun4* mutant in *Chlamydomonas*. Although this is not the first article to report on *gun4* mutant, it is important as it does provides extra phenotypic studies that show both consistent results and novel investigations. I do have some minor concerns that  the authors  should consider to improve the manuscript:

**1.** The title is somewhat misleading as the progressive Chl deficiency is just one of the phenotypes of this mutant; please consider properly revising it.

**2.** Why did the authors need to put up so many figures in one article? Some of the figures are not really necessary and can be put attached online as supplementary figures. With too many figures, the article can become very boring to read.

**3.** The authors should put some more emphasis on the interesting findings like the genetic rearrangement of the gun4 gene in the mutant, for example.

**4.** Unless the authors made further characterization on the two HYP genes regarding their sequences and deduced amino acid and possible functions, they can be omitted from the paper as they would confuse the reader.

I have read this submission. I believe that I have an appropriate level of expertise to confirm that it is of an acceptable scientific standard.
